# Next Generation Orthopaedic Implants by Additive Manufacturing Using Electron Beam Melting

**DOI:** 10.1155/2012/245727

**Published:** 2012-08-21

**Authors:** Lawrence E. Murr, Sara M. Gaytan, Edwin Martinez, Frank Medina, Ryan B. Wicker

**Affiliations:** ^1^Department of Metallurgical and Materials Engineering, The University of Texas at El Paso, El Paso, TX 79968, USA; ^2^W.M. Keck Center for 3D Innovation, The University of Texas at El Paso, El Paso, TX 79968, USA

## Abstract

This paper presents some examples of knee and hip implant components containing porous structures and fabricated in monolithic forms utilizing electron beam melting (EBM). In addition, utilizing stiffness or relative stiffness versus relative density design plots for open-cellular structures (mesh and foam components) of Ti-6Al-4V and Co-29Cr-6Mo alloy fabricated by EBM, it is demonstrated that stiffness-compatible implants can be fabricated for optimal stress shielding for bone regimes as well as bone cell ingrowth. Implications for the fabrication of patient-specific, monolithic, multifunctional orthopaedic implants using EBM are described along with microstructures and mechanical properties characteristic of both Ti-6Al-4V and Co-29Cr-6Mo alloy prototypes, including both solid and open-cellular prototypes manufactured by additive manufacturing (AM) using EBM.

## 1. Introduction

Although Ti and its alloys have been used for more than half a century as monolithic, solid implant materials, they are limited by lack of fusion and bone resorption due to stress shielding. This results from an order-of-magnitude greater metal stiffness (or Young's modulus) relative to cortical (hard) bone and up to two orders of magnitude for cancellous (or trabecular), soft bone [1–3]. Wear debris production for contacting surfaces and the elimination of necessary vascularization are also often attendant issues [4]. However, the presence of a nonporous, stable passive film (TiO_2_) on the surface minimizes the diffusion of metal ions from the bulk material and prevents corrosion of the material in contact with human tissues [2]. Other metallic alloys such as stainless steel (316L) and Co-Cr (or Co-Cr-Mo) alloys are also used, especially in preference to Ti alloys for load-bearing applications due to limited strength or poor fatigue properties, and critical wear applications. These alloys also rely on the presence of chromium for their corrosion resistance. However, breakdown of passivating layers, variations in the physiological environment, including infection, can increase corrosion or corrosion rate as well as corrosion products. Consequently, biocompatibility in its broadest sense is a complex issue [1, 2, 5].

While conventional orthopaedic knee and hip implants in particular, fixed with acrylic cement, have produced excellent results in older patients, less success is generally achieved for younger, more active individuals [7]. As alternatives to acrylic cement as well as other benefits promoting biocompatibility, porous scaffolds have exhibited considerable potential because in addition to promoting bone cell ingrowth for implant stabilization, porosity or cellular density variations can allow for stiffness selections to better match the modulus of different bone types. Unfortunately, only porous-coated implant applications have been attempted, and these appliances often suffer from the fact that initial stabilization requires precise bone press-fit to initiate tissue ingrowth. These surface coatings are also prone to cracking under fatigue conditions, detachment, granulation, and electrochemical incompatibility where dissimilar metal or alloy coatings are employed. Metal and alloy cellular structures, including foams, are difficult to produce as a consequence of their high melting/sintering temperatures and chemical reactivity. Even more challenging, however, is the ability to fabricate monolithic orthopaedic appliances with requisite porosity or varying (and functional) porosity or cellular density [5, 8, 9]. Cellular in this context might be envisioned as a foam, for example.

Additive manufacturing (AM) using electron beam melting (EBM) has recently illustrated not only the potential for fabricating complex, porous, monolithic implant components but also the prospect of fabricating patient-specific implant components. This paper reviews progress and potential advances to be made in the EBM fabrication of Ti-6Al-4V and Co-29Cr-6Mo alloy implant prototypes, especially total knee, hip, and novel intramedullary rod development [10–13].

## 2. Fabrication, Testing, and Characterization Methods

### 2.1. EBM System Principles

**Figure 1:**
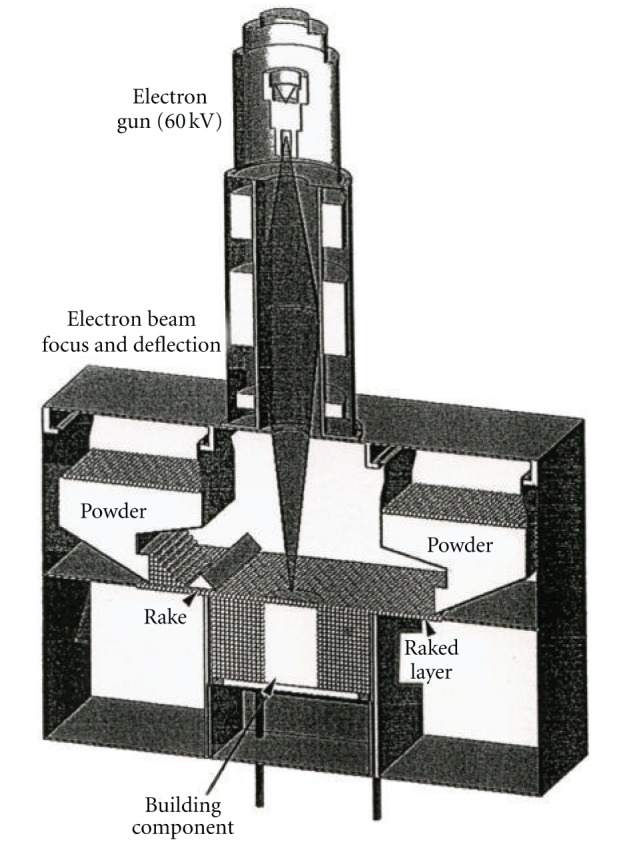
Schematic view of the Arcam A2 electron beam melting (EBM) system.

Electron beam melting (EBM) as an additive (layer) manufacturing platform has been commercially available for a decade from Arcam AB, Sweden.  Figure 1 illustrates a simple schematic view for the Arcam A2 EBM system used in much of the work to be described herein. The system is basically an electron optical column where an electron beam is generated, focused, and scanned (or rastered) over a uniformly raked powder layer which is gravity fed from cassettes shown. Each layer (~50 to 100 *μ*m thick) is preheated to temperatures ranging from 600 to 800°C using multiple beam passes at scan rates >10^4^ mm/s at high current, followed by a melt scan at reduced scan rate and beam current (>10^2^ mm/s; <10 mA). The melt scan is selectively driven by a 3D-CAD (computer-aided design) software model which melts only selected layer portions which are added in the building direction to create complex, 3D structures in a vacuum chamber which can accommodate building components up to ~0.2 m^3^. Process optimization involves beam focus selection and melt scan beam current depending upon the powder properties, especially melting point.

In this work, orthopaedic implant prototypes have been fabricated from medical grade Ti-6Al-4V-ELI (extra low interstitial) powder having a nominal composition of 6.04% Al, 4.05% V, 0.013% C, 0.0107% Fe, 0.13% O, and the balance Ti-(in weight percent). The average powder size was ~30 *μ*m, and the powder had a melting temperature of ~1630°C. Prototype implant components were also fabricated from ASTM-F75 Co-base powder having a nominal composition of 29% Cr, 6% Mo, 0.22% C, 0.25% Ni, 0.7% Si, and the balance Co-(in weight percent). The average powder size was ~40 *μ*m, and the powder melting point was ~1430°C.

### 2.2. Cellular System Models

**Figure 2:**
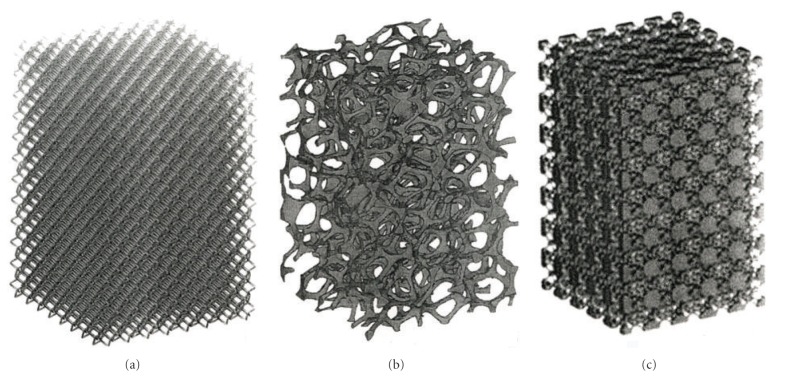
Examples of open-cellular structure CAD models for additive manufacturing using EBM. (a) Dode-thin unit cell/element lattice-mesh structure. (b) Foam structure. (c) Bone unit cell/element lattice structure (http://www.pro-fit.de/).

Complex, open-cellular CAD models employing accessible software design elements have been described in some detail elsewhere [13]. These models utilize simple unit cell or geometrical elements which can be linearly expanded into 3D lattice structures having adjustable strut or ligament dimensions and spacings to control the porosity or density. These design strategies can be integrated into monolithic layer building in the EBM. CT and related digital layer scanning can also be used to create CAD models for layer building of complex 3D prototypes.  Figure 2 illustrates several regular mesh and foam models utilized in this study for fabricating porous (varying density) orthopaedic component prototypes using Ti-6Al-4V and Co-29Cr-6Mo alloy powders in the EBM system (Figure 1).

**Figure 3:**
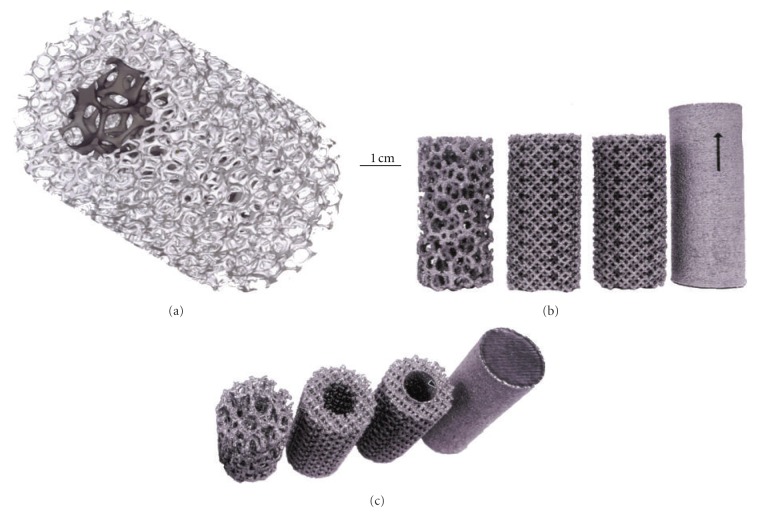
Monolithic cellular/solid structures. (a) CAD model for porous foam core surrounded by less porous (higher density) foam structure. (b) Ti-6Al-4V components fabricated by EBM. (c) Alternative, perspective view of (b). Arrow shows a thin, solid tube core surrounded by foam structure. The arrow for the solid cylinder in (b) shows the build direction parallel to the cylinder axis.

Figure 3(a) shows a foam cylinder model having an inner, lower density foam stem surrounded by a higher-density foam shell, which might suggest inner and outer femoral bone structure. Figures 3(b) and 3(c) illustrate a range of Ti-6Al-4V EBM-fabricated components utilizing the CAD models shown in Figures 2(a) and 2(b), with different perspectives.

### 2.3. Dynamic Stiffness Measurements for Open-Cellular Structures

Open cellular structures, especially foams, can have significantly reduced stiffnesses or Young's modulus with corresponding reductions in density and are generally described by Gibson and Ashby [14] in the form

(1) E=Eo(ρρo)n,

where *E* is the stiffness for an open-cellular structure having a density *ρ* and *E*_*o*_ and *ρ*_*o*_ are the corresponding solid (fully dense) stiffness and density, respectively. For Ti-6Al-4V *E*_*o*_ = 110 GPa, *ρ*_*o*_ = 4.43 g/cm^3^. For Co-29Cr-6Mo alloy, *E*_*o*_ = 210 GPa, *ρ*_*o*_ = 8.44 g/cm^3^ [13]. For a wide range of aluminum and aluminum alloy foams, *n* in (1) has been shown to vary from ~1.8 to 2.2 [15], while recent studies of other metal and alloy foams (including Ti-6Al-4V, Cu, and Co-29Cr-6Mo) [13, 16, 17] have exhibited similar values of *n* (2.0 to 2.3). As a general rule of thumb, *n* has often been assumed to be 2.

Dynamic stiffness can be conveniently measured in a nondestructive test which utilizes a resonant frequency or vibration mode established by systematic tapping of an optimum specimen size according to the expression [13, 18]

(2) E=ςmfr2,

where *E* is the Young's modulus or dynamic stiffness number, *ς* is a specimen shape factor, *m* is the specimen mass, and *f*_*r*_ is the resonant frequency. The test specimen shape is dictated by general foam requirements established by Ashby et al. [15].

### 2.4. Characterization of Microstructural and Mechanical Behavior

 It is already well established that the microstructure of Ti-6Al-4V laser or electron beam fabricated components is influenced by the build parameters and build geometry, as these control the solidification and cooling rates. Selective laser melting (SLM) utilizes a much more rapid melt scan rate than EBM, and most SLM Ti-6Al-4V microstructures are dominated by *α*′-martensite in contrast to more conventional acicular *α*-phase platelets [13, 19, 20]. This is also true for EBM-fabricated mesh and foam structures of Ti-6Al-4V where solidification and cooling are significantly different from solid components (Figure 3(b)) [13]. Other unusual, columnar microstructures influenced by directional solidification have also been observed for the EBM fabrication of Co-29Cr-6Mo alloy components, including mesh structures [21]. Since microstructure-property relationships generally determine materials performance, it is important to characterize both the microstructures (or microstructural evolution) and the corresponding mechanical behavior for EBM-fabricated prototypes, especially open-cellular structures.

The details for observing *α*-phase and *α*′-martensite microstructures in EBM-fabricated Ti-6Al-4V solid and open-cellular structures by optical metallography have been described in detail elsewhere [13, 19]. These microstructures rely upon mounting, polishing, and selective etching using varying concentrations of hydrofluoric and nitric acids in water and varying times of etchant exposure. Open cellular mesh and foam structures such as those shown in Figure 3(b) are mounted with specific orientations for imaging, including the build direction. Similar preparation and imaging were performed for Co-29Cr-6Mo solid and open-cellular components utilizing etchants consisting of varying concentrations of hydrochloric and nitric acids in water, with ferrous chloride additions, or hydrochloric acid/hydrogen peroxide mixtures [21].

Because of the difficulty in producing thin films from mesh struts and foam ligaments, these related microstructures and substructures can be compared with solid, bulk microstructures utilizing optical metallography and corresponding transmission electron microscope (TEM) observations [13, 21]. Mesh and foam strut and ligament surface features were also observed by scanning electron microscopy (SEM), and these observations were compared to optical metallograph images connecting the interior microstructures for EBM components to the surface structures and surface roughness features.

The difficulty in extending solid component mechanical properties to open-cellular structures is similar to the microstructural comparisons noted previously. Unlike solid structures, ideal cellular structures exhibit simultaneous yielding and collapse resulting in a distinct yield strength coincident with a plateau of flow stress. Open cellular structures are susceptible to bending causing their stiffness to be subject to the scaling illustrated in (1) [14]. However, yield stress for struts or ligaments can be estimated from microindentation (Vickers) hardness measurements (HV) on these structures in mounted specimens, since as a rule of thumb the yield strength is ~HV/3. Correspondingly, solid specimen tensile data for components fabricated with different solidification and cooling strategies can be compared with their associated microstructures, and these structure-property relationships were compared with microindentation hardness measurements for solid and open-cellular structures having similar microstructures. Solid Ti-6Al-4V and Co-29Cr-6Mo alloy tensile and hardness data (including Vickers microindentation hardness (HV) and Rockwell C-scale macroindentation hardness (HRC)) have been summarized for reference and comparison for EBM solid cylinders (as in Figure 3(b)) as shown in Table 1 [13, 17, 21].

## 3. Results and Discussion

### 3.1. Total Knee Implant Prototypes

**Figure 4:**
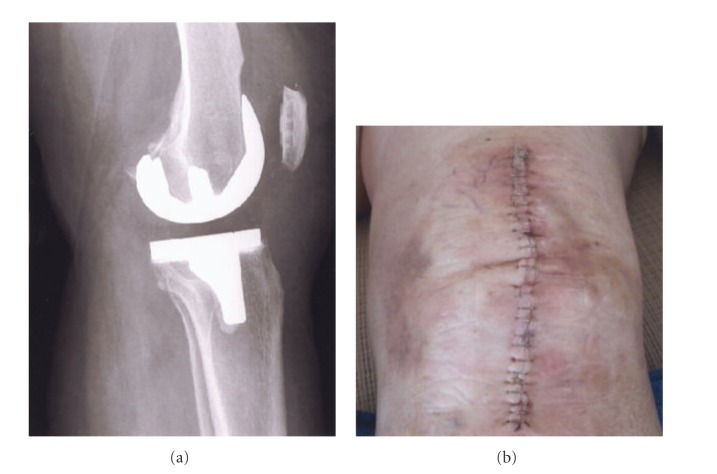
X-ray image (a) and incision-suture photograph for total knee replacement (b) (Courtesy of Patricia Murr).

**Figure 5:**
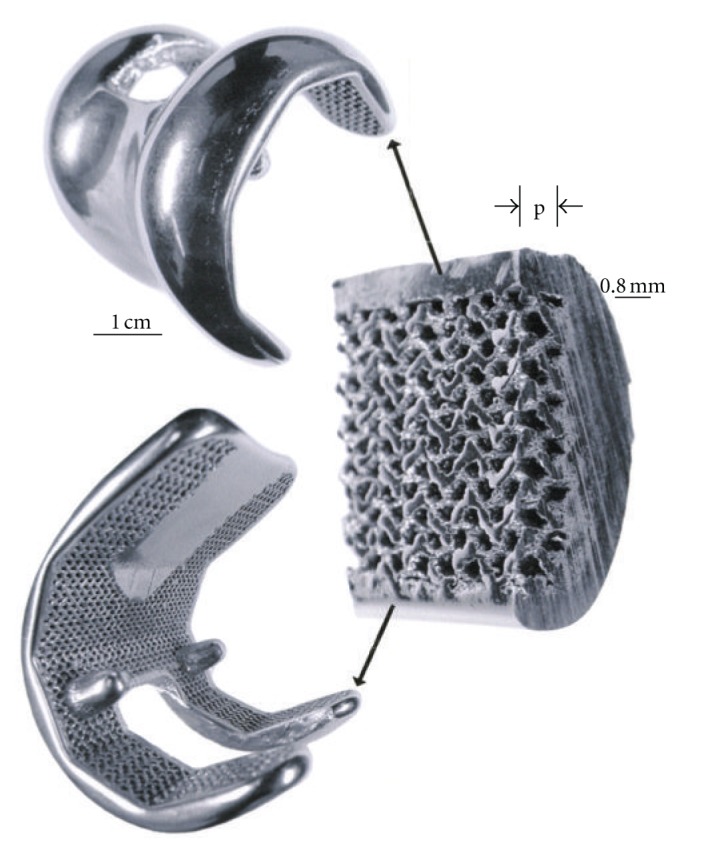
Co-29Cr-6Mo alloy femoral knee implant prototype fabricated by EBM and HIPed according to ASTM-F75 standard. The insert illustrates the porous inner surface zone (p) composing the monolith.

**Figure 6:**
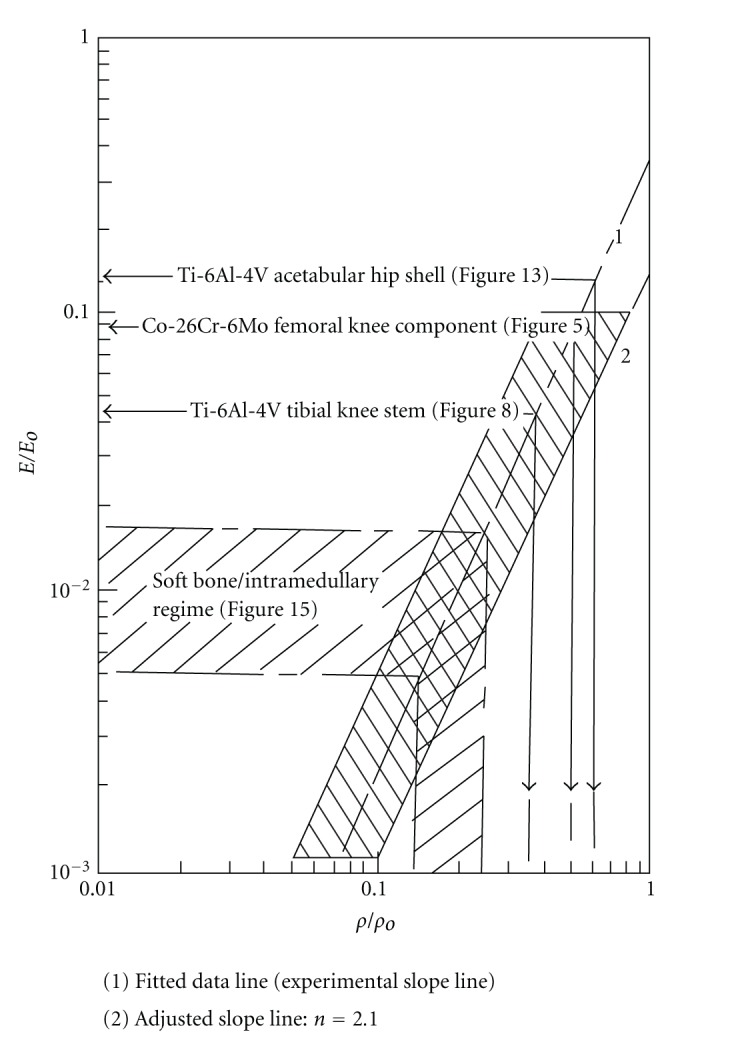
Stiffness-density design plot composed of experimental data [6]. The data field for Ti-6Al-4V and Co-29Cr-6Mo is shown shaded. The shaded regime follows a fitted slope, *n* = 2.1. Examples for prototype designs discussed in the text are noted.

Knee implants commonly utilize a cast Co-29Cr-6Mo alloy femoral appliance while the tibial stem and meniscal platform are wrought Ti-6Al-4V. The Co-29Cr-6Mo alloy is harder and more wear resistant and is cemented to the end of the prepared femur, while the Ti-6Al-4V implant is commercially fabricated with varying tibial stem sizes. In severe circumstances, the femoral component can also employ a stem which is cemented into the femoral core.  Figure 4 illustrates these features with a knee replacement X-ray image along with a frontal view of the closed incision for the surgical replacement. It can be appreciated that the femoral appliance in particular can benefit from a porous surface or surface region to allow for efficient femoral bone cell ingrowth and how surface coatings of various kinds would be compromising and prone to failure considering the complexity and magnitude of the bearing stresses. This situation presents a unique fabrication prospect for EBM since a fairly rigid and sufficiently porous structure can be built as a functional monolithic implant as illustrated in Figure 5. The inner cellular mesh structure shown in Figure 5 can be varied as necessary for adequate bone ingrowth, strength, and density, since the density will determine the effective stiffness and prospects for eliminating stress shielding using relative stiffness versus relative density design plots as illustrated in Figure 6 for both Co-29Cr-6Mo and Ti-6Al-4V alloy open-cellular structures. The stiffness-density design plot shown in Figure 6 was constructed from experimental data for Co-29Cr-6Mo and Ti-6Al-4V, having a corresponding slope (*n*) in (1) of 2.1 [6]. In the Co-29Cr-6Mo open cellular mesh structure shown in Figure 5, the relative density was estimated to be 0.5 based upon the strut and cellular dimensions, corresponding to an estimated relative stiffness of 0.09 in Figure 6 or a stiffness matching the contacting femur end estimated to be 19 GPa for *E*_*o*_ = 210 GPa.

**Figure 7:**
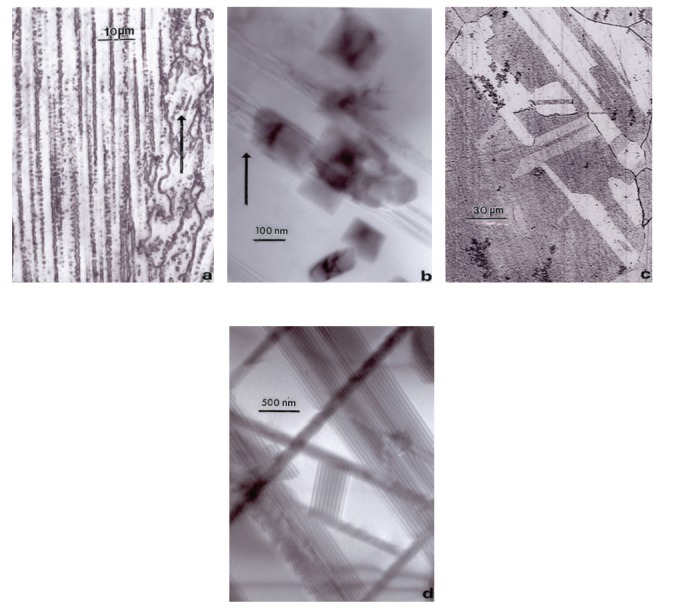
Comparative microstructures for Co-29Cr-6Mo components. (a) Optical micrograph showing columns of Cr_23_C_6_ precipitates in the build direction (arrow). (b) TEM image of Cr_23_C_6_ precipitate columns in (a). (c) Optical micrograph showing HIP-annealed grain structure. (d) TEM image for intrinsic stacking faults in (c).

The EBM-fabricated Co-29Cr-6Mo femoral component shown in Figure 5 was HIPed according to the ASTM-F75 standard after fabrication, finished, machined, and polished. HIPing involved an anneal at 1200°C for 4 h and an additional homogenization at 1220°C for 4 h. This altered the microstructure and mechanical properties as illustrated in Figure 7 and Table 1. Figures 7(a) and 7(b) show the columnar Cr_23_C_6_ precipitate architecture which characterizes the as-fabricated, EBM components, including both solid and open-cellular structures as described in detail by Gaytan et al. [21]. After HIP and anneal, these carbide columns dissolve, forming an equiaxed fcc grain structure containing {111} coherent annealing twins and a notable density of intrinsic stacking faults as shown for comparison in Figures 7(c) and 7(d). Table 1 shows the hardness and tensile properties for the ASTM-F75 standard and the as-fabricated EBM (*z*) component (cylindrical standard built in the *z*-axis direction perpendicular to the additive layers). While the hardness (HRC) and UTS are much higher for the EBM-fabricated product, the elongation is only 3.6% in contrast to ~10% for the ASTM F75 HIP standard for wrought alloy. However, the HIPed and annealed EBM (*z*) component (EBM (*z*) + HIP) has a higher hardness, tensile yield stress (YS), ultimate tensile stress (UTS), and a much greater elongation than the ASTM-F75 standard. The elongation for the EBM (*z*) + HIP product has an elongation roughly an order of magnitude greater than the as-fabricated component (EBM (*z*) in Table 1). This is primarily a consequence of the microstructure variation illustrated in comparing Figure 7.

While Table 1 does not include corresponding hardness values for the open-cellular Ti-6Al-4V (Figure 3) or Co-29Cr-6Mo components (represented conceptually in Figures 2 and 3), Ti-6Al-4V mesh-strut and foam ligament hardness (HV), were observed to range from 4.5 to 5.2 GPA. Correspondingly, the Co-29Cr-6Mo alloy mesh-strut and foam ligament hardnesses varied from 4.5 to 6 GPa, with the higher hardness ranges associated with the smallest diameter struts or ligaments where the cooling rates were highest.

**Figure 8:**
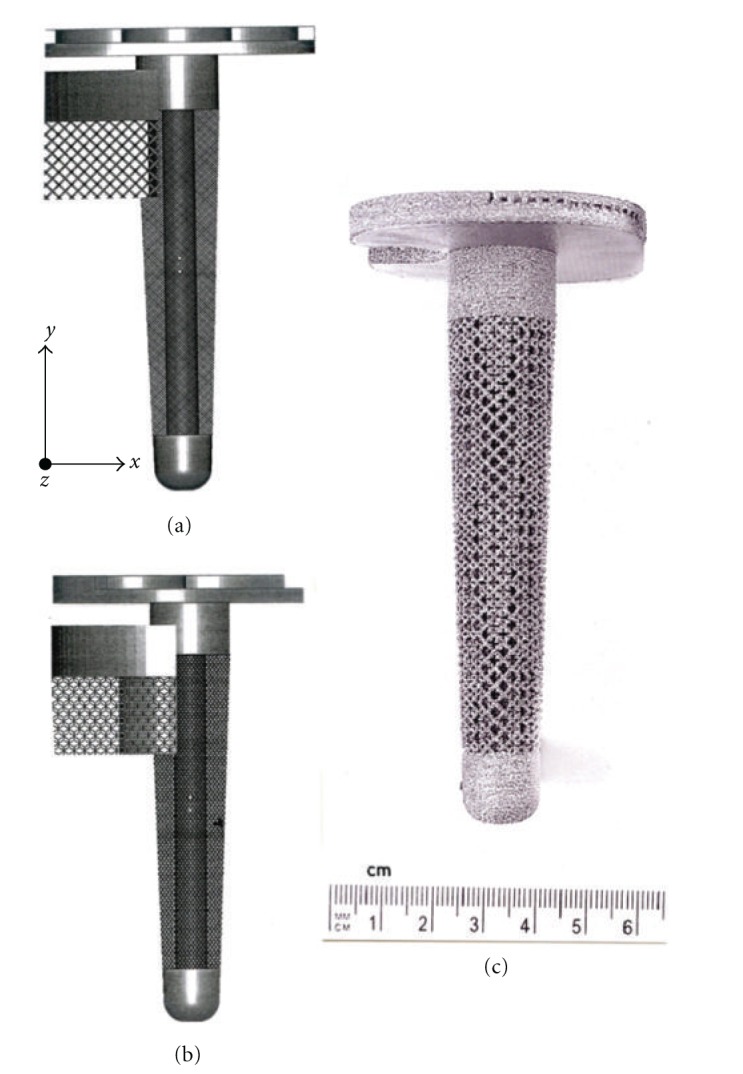
Tibial (knee) Ti-6Al-4V stem, monolithic prototype development example. (a) CAD model orientation with respect to the solid stem (core) axis. Mesh structure is based on dode-thin element in Figure 2(a). (b) EBM-fabricated prototype using CAD model in (a). (c) CAD model in (a) rotated 45°.

In contrast to a high stiffness for the contacting Co-29Cr-6Mo femoral appliance in Figure 5, a Ti-6Al-4V tibial stem appliance might have an estimated stiffness of 5 GPa if it is not a press fit with the outer, cortical bone of the tibia. Consequently, utilizing a corresponding relative stiffness of 0.045 (and considering *E*_*o*_ = 110 GPa for Ti-6Al-4V), the relative design density and actual mesh density can be estimated from Figure 6 to be 0.35 and 1.55 g/cm^3^, respectively.  Figure 8 shows a longer Ti-6Al-4V tibial stem having a solid core and an outer mesh structure based on the CAD model illustrated in Figure 2. In contrast to Figure 4, the longer stem with more compatible stiffness may provide additional stability for the implant. Long stems have in fact also been used for femoral bone damage to connect the Co-Cr-Mo appliance (Figure 5) to the femur as noted previously. Moreover, the solid central core can also be replaced by a cellular structure having a more compatible stiffness and functionality, either using mesh or foam arrays or combinations as implicit in Figure 3.

**Figure 9:**
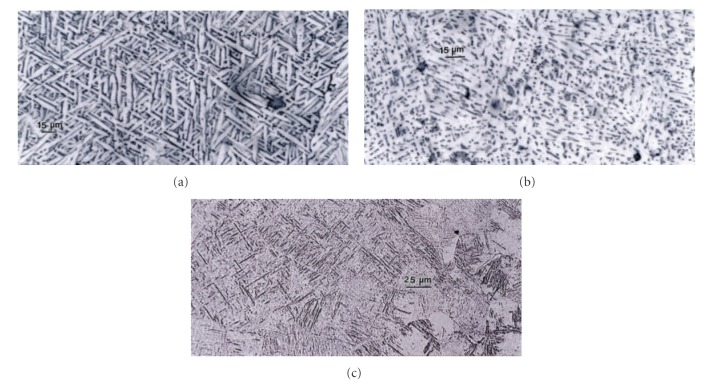
Comparison of Ti-6Al-4V solid, cylindrical component microstructures. (a) EBM (*z*) component *α*-phase (acicular) plates. (b) EBM (*x*, *y*) component fine *α* + *β* structure. (c) *α* + *α*′ martensite structure for mesh component as in Figure 8(b). Corresponding Vickers microindentation hardness values are (a) 3.5 GPa; (b) 4.1 GPa; (c) 4.5 GPa.

Figure 9(a) shows the microstructures for an EBM-fabricated cylinder in the build direction or along the *z*-axis, while Figure 9(b) compares the microstructure for a cylinder fabricated along the *x* or *y* axis (or direction) and perpendicular to the build direction (Figure 3). A typical mesh or foam strut or ligament microstructure is shown in Figure 9(c). Figure 9(a) shows *α*-phase acicular plates with dark *β* (bcc) boundaries or boundary phase, having an average width of ~6 *μ*m, while Figure 9(b) shows a finer, mixed *α* + *β* phase microstructure. In Figure 9(c), the microstructure is primarily *α*′-martensite plates with an average *α*′ plate width of ~2 *μ*m. The corresponding Vickers microindentation hardness values are shown in the caption for Figure 9 as well. It is apparent that the finer *α*′-martensite contributes to a higher hardness, and this is somewhat fortuitous since it may be desirable to have hard, strong mesh or foam structures to optimize the performance of these stiffness-compatible implants. Since the yield stress can be estimated from the Vickers microindentation hardness, the corresponding specific strengths (YS/*ρ*) for the three Ti-6Al-4V build components in Figure 9 are observed to be 1.2, 1.4, and 1.5 GPa-cm^3^/g, considering YS (yield stress) ~HV/3 and assuming a mesh or foam density in Figure 9(c) to be ~1 g/cm^3^.

**Figure 10:**
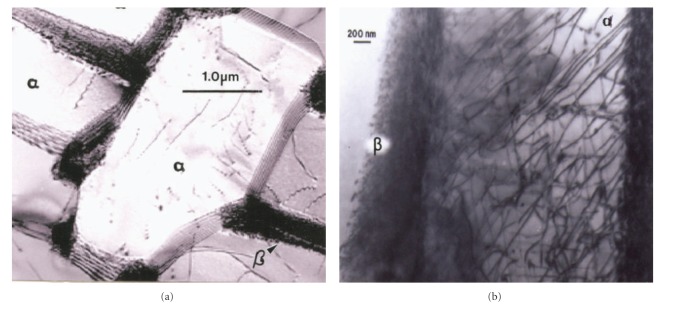
TEM image comparison for dislocation substructures in EBM-fabricated Ti-6Al-4V components as in Figure 9(a). (a) Low dislocation density and shallow *β* wall thickness surrounding *α*-phase grains. (b) High dislocation density and thicker *β* phase surrounding *α*-grains. Hardness in (a) and (b) was HV 3.6 and 3.9 GPa, respectively.

The cooling rate for Ti-6Al-4V builds can also influence the residual dislocation density by more than an order of magnitude, and this feature is shown for *α*-phase plate structure or substructure in Figure 10. Variations in microindentation hardness corresponding to Figures 10(a) and 10(b) have average values (HV) of ~3.6 and 3.9 GPa, respectively, a reflection of the distinct difference in dislocation structures on comparing Figures 10(a) and 10(b).

### 3.2. Total Hip Implant Prototypes

**Figure 11:**
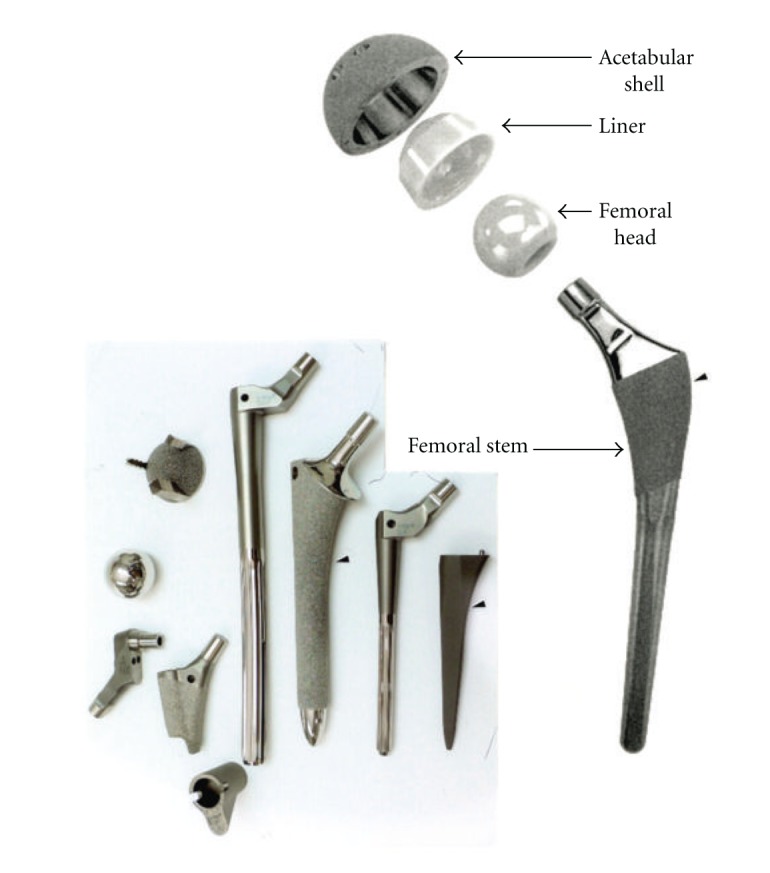
Total hip replacement components and examples of commercial hip stem implants. Small arrows illustrate porous coating areas using sinter technologies. Commercial examples (lower left) courtesy of DiSanto, Inc.

**Figure 12:**
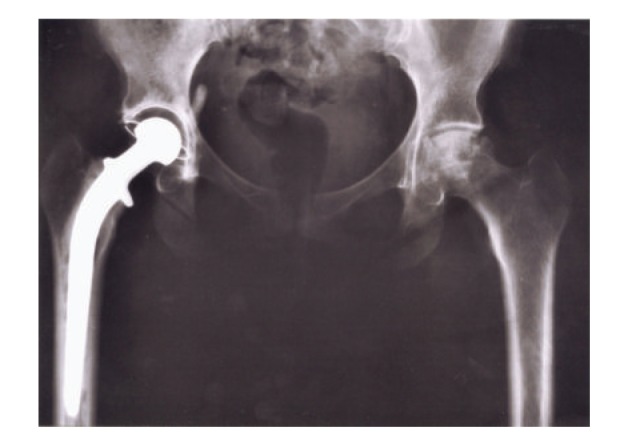
Hip X-ray image showing total replacement at left (right side) (from Wikipedia).

**Figure 13:**
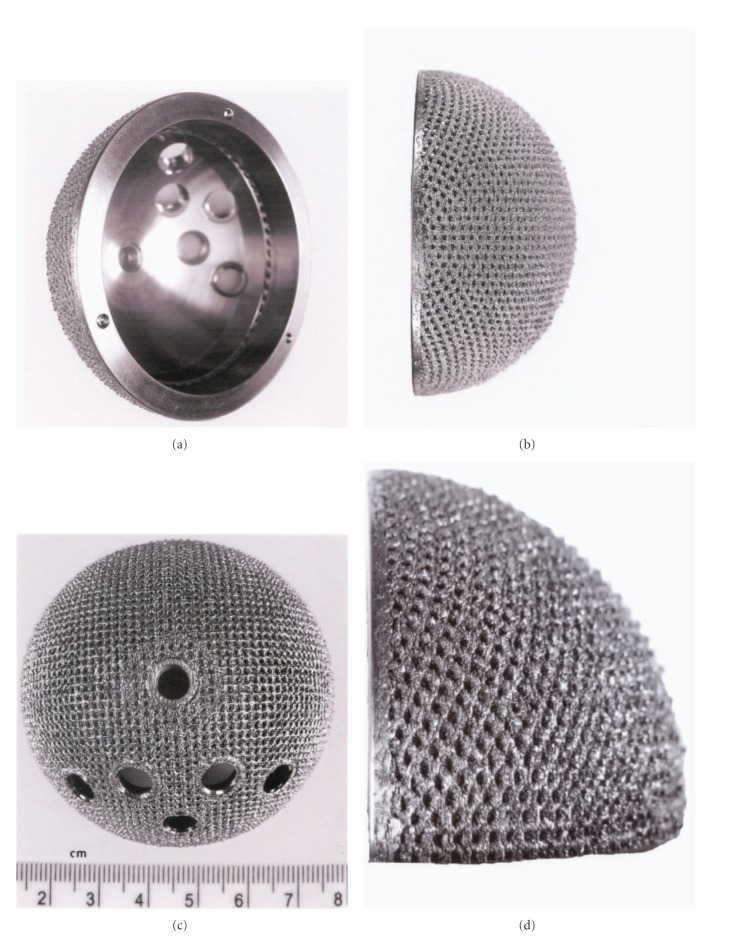
EBM-fabricated Ti-6Al-4V acetabular cup with outer porous-mesh structure region for hip bone (socket) ingrowth. (a) and (b) show rotated views while (c) and (d) show magnified sections illustrating the porous-mesh structure (sample courtesy of Arcam).

**Figure 14:**
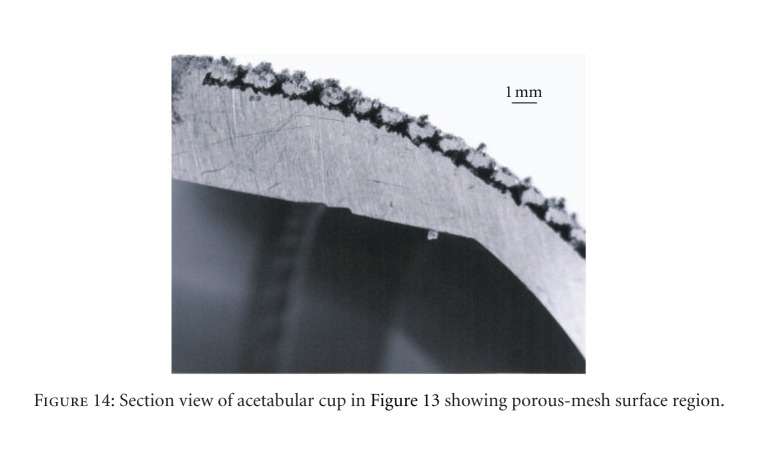
Section view of acetabular cup in Figure 13 showing porous-mesh surface region.

Figure 11 shows the total hip replacement components along with several examples of commercial hip (femoral) stem components. These components are primarily Ti-6Al-4V alloy. A portion of most hip stems contain a porous coating, often consisting of sintered beads which provide a relatively shallow coating with a limited, effective porosity. The acetabular shell (or cup), which is placed in the hip socket as shown in the X-ray image in Figure 12, has recently been produced commercially by EBM. This has allowed for the fabrication of a monolithic component with an open-cellular, porous outer structure as illustrated in the series of photographs in Figure 13. The outer, open-cellular (mesh) surface (Figure 14) has an approximate relative density of 0.6 similar to the femoral knee component in Figure 5 and corresponds to a relative stiffness from Figure 6 of ~0.13 or a porous, transitional-region stiffness of ~14 GPa. Although limited in thickness, the porous region can allow for some bone cell ingrowth, with a pore size of ~1 mm. The microstructure for the solid thickness section for the acetabular cup shown in Figure 13(d) was similar to the *α*-phase acicular plate microstructure shown in Figure 9(a), with some fine *β* as indicated in the more rapidly cooled horizontal Ti-6Al-4V EBM-fabricated component shown in Figure 9(b). This mesh structure is shown in Figure 14.

**Figure 15:**
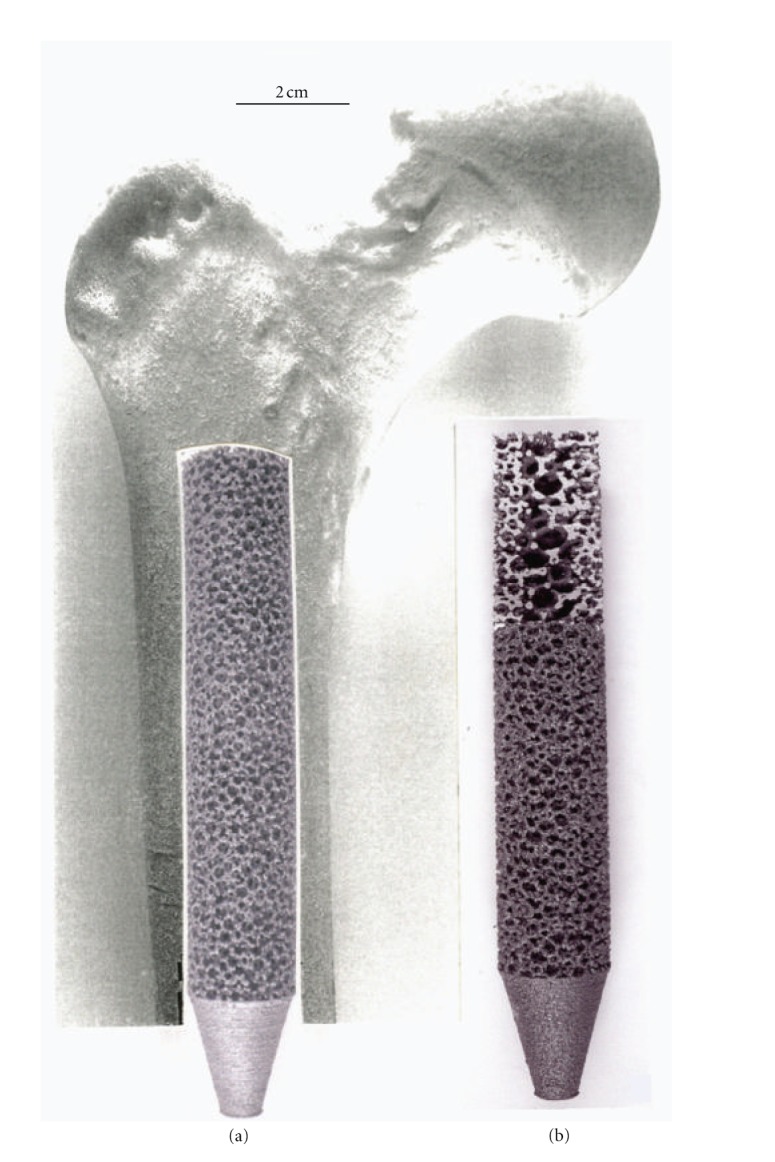
Upper femur with simulated intramedullary rod insert fabricated from Ti-6Al-4V by EBM in (a). (b) shows a through-section cutaway view illustrating inner, more porous foam core surrounded by more dense foam structure for stiffness compatibility.

**Figure 16:**
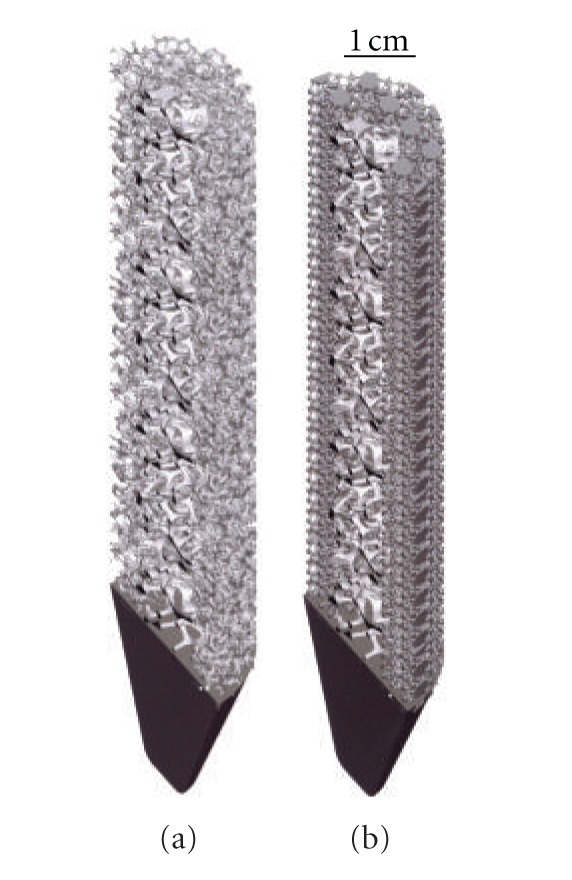
CAD-model examples of intramedullary rod or hip stem prototypes. (a) Section view for foam of higher density (and stiffness) surrounding foam of lower density (and stiffness) as in Figure 15(b). (b) Section view for foam of low-density core surrounded by high-density mesh (bone element in Figure 2(c)) structure.

Just as the tibial knee stem can be fabricated as a porous-monolithic appliance by EBM, femoral hip stems as shown commercially in Figure 11 can also be similarly fabricated. In addition, intramedullary rods might benefit from the ability to develop functionally graded or outer, porous structures which can more closely match the harder, cortical femoral bone stiffness. Consequently, intramedullary rods or femoral hip stems (Figure 11) can be fabricated with a central solid rod and an outer mesh or foam structure, as illustrated in Figure 8. However, the interior femoral (hip) stem could be a more open cellular mesh or foam, having a stiffness more compatible with the trabecular or soft, intramedullary regime, and this design may also ideally promote vascularization.  Figure 15 illustrates this concept for a Ti-6Al-4V rod fabricated with a lower-density central foam core (with a density of ~0.6 g/cm^3^) and an outer foam structure with a density of ~1.1 g/cm^3^. This corresponds to an outer foam stiffness of 2.2 GPa and an ideal inner foam core stiffness of 0.3 GPa (utilizing the relative stiffness-density design plots in Figure 6).  Figure 16 shows CAD models combining different density foam structures as fabricated in Figure 15, which can serve as a rod model section composed of a central foam core and an outer foam or mesh-type structure having a higher stiffness (Figures 16(a) and 16(b), resp.), as described above.

As discussed previously, the mesh and foam arrays fabricated by EBM possess an intrinsic hardness which is a result of the rapid cooling these structures experience. Consequently, they experience a high specific strength as defined by YS/*ρ* or HV/3*ρ*. These values can range from 0.8 and 0.4 GPa · cm^3^/g for Ti-6Al-4V and Co-29Cr-6Mo alloy, respectively, to >6 GPa · cm^3^/g, roughly an order of magnitude higher for porous (open-cellular) structures than solid structures. However, these implant prototypes have not been mechanically and physiologically tested, and there is no specific or comparative data for these conceptual implant components. In particular, there is no comparative fatigue data for the open-cellular structures, especially in the implant component context.

## 4. Closure

While we have demonstrated only a few examples of additive manufacturing using EBM to fabricate orthopaedic implants involving knee and hip replacements, the prospects of fabricating such replacement components on a patient-specific basis are even more promising. While these examples represent only a few concepts involving complex monolithic implants, fatigue measurements of the open-cellular structures, their accommodation of bone cell ingrowth, and prospects for vascularization will provide the foundation for new orthopaedic innovations promoting implant compatibility and dependability.

It is of interest to note that there have been at least 1000 acetabular shells as illustrated in Figure 13, fabricated by EBM, finished, and implanted in humans over the past several years, with considerable success. Ala Ortho of Italy received European CE certification in 2007 and has manufactured these acetabular hip shells as so-called Fixa Ti-Por cups from Ti-6Al-4V as illustrated in Figure 13. Additionally, Harrysson and Cormier [22] have also recently discussed the prospects for custom orthopaedic implants, including their cost effectiveness especially regarding compatibility and material and manufacturing savings in contrast to commercial, wrought products.

## Figures and Tables

**Table 1:** Comparison of mechanical properties for solid Ti-6Al-4V and Co-26Cr-6Mo.

Material	Microstructure	HV (GPa)	HRC	YS (GPa)	UTS (GPa)	Elongation (%)
Wrought Ti-6Al-4V	Coarse acicular *α*-phase	3.8	48	1.17	1.23	12
EBM (*z*) Ti-6Al-4V	Acicular *α*-phase	3.6	38	1.03	1.11	13
EBM (*x*,*y*) Ti-6Al-4V	*α*′-martensite + *α*-phase	4.1	41	1.10	1.11	11
ASTM Grade 5 (nominal) Ti-6Al-4V	Acicular *α*-phase plates	3.4	37	0.9	1.00	15
ASTM-F75 (wrought) Co-26Cr-6Mo	Equiaxed grains with annealing twins	2.8	30	0.45	0.66	10
EBM (*z*) Co-26Cr-6Mo	Columnar precipitates (Cr_23_C_6_)	4.6	48	0.51	1.45	3.6
EBM (*x*,*y*) Co-26Cr-6Mo	Columnar precipitates (Cr_23_C_6_)	—	—	0.77	0.84	2.7
EBM (*z*) + HIP Co-26Cr-6Mo	Equiaxed fcc grains with annealing twins	3.6	31	0.60	1.15	32
EBM (*x*,*y*) + HIP Co-26Cr-6Mo	Equiaxed fcc grains with annealing twins	—	—	0.63	0.99	20
